# The Use of Animal Models to Decipher Physiological and Neurobiological Alterations of Anorexia Nervosa Patients

**DOI:** 10.3389/fendo.2015.00068

**Published:** 2015-05-19

**Authors:** Mathieu Méquinion, Christophe Chauveau, Odile Viltart

**Affiliations:** ^1^INSERM UMR-S1172, Development and Plasticity of Postnatal Brain, Lille, France; ^2^Pathophysiology of Inflammatory Bone Diseases, EA 4490, University of the Littoral Opal Coast, Boulogne sur Mer, France; ^3^INSERM UMR-S1172, Early stages of Parkinson diseases, University Lille 1, Lille, France

**Keywords:** genetic models, environmental models, anorexia nervosa, acute stress, social stress, food restriction, activity/hyperactivity

## Abstract

Extensive studies were performed to decipher the mechanisms regulating feeding due to the worldwide obesity pandemy and its complications. The data obtained might be adapted to another disorder related to alteration of food intake, the restrictive anorexia nervosa. This multifactorial disease with a complex and unknown etiology is considered as an awful eating disorder since the chronic refusal to eat leads to severe, and sometimes, irreversible complications for the whole organism, until death. There is an urgent need to better understand the different aspects of the disease to develop novel approaches complementary to the usual psychological therapies. For this purpose, the use of pertinent animal models becomes a necessity. We present here the various rodent models described in the literature that might be used to dissect central and peripheral mechanisms involved in the adaptation to deficient energy supplies and/or the maintenance of physiological alterations on the long term. Data obtained from the spontaneous or engineered genetic models permit to better apprehend the implication of one signaling system (hormone, neuropeptide, neurotransmitter) in the development of several symptoms observed in anorexia nervosa. As example, mutations in the ghrelin, serotonin, dopamine pathways lead to alterations that mimic the phenotype, but compensatory mechanisms often occur rendering necessary the use of more selective gene strategies. Until now, environmental animal models based on one or several inducing factors like diet restriction, stress, or physical activity mimicked more extensively central and peripheral alterations decribed in anorexia nervosa. They bring significant data on feeding behavior, energy expenditure, and central circuit alterations. Animal models are described and criticized on the basis of the criteria of validity for anorexia nervosa.

## Introduction

Eating disorders represent a large field of investigation in industrialized societies where food intake behaviors and quality of food become indisputable and incoherent. Research projects are currently focused on obesity, a dramatic consequence of overconsumption of fat and carbohydrates. However, populations of these societies also suffer of other dramatic but underinvestigated eating disorders. These eating disorders are presently defined according to the American Manual of Psychiatry DSM-5 (1) and divided into three main subtypes of eating disorders: anorexia nervosa (AN), bulimia nervosa (BN), and binge eating disorder (BED). The main characteristics of these three subtypes are summarized in Table 1. As usually mentioned by psychiatrists, the subtype determination at the time of diagnosis should be considered carefully since the majority of women with AN crossed over between the other subtypes [BED or BN; (2)].

**Table 1:** **Main characteristics of the mean eating disorders: anorexia nervosa (AN), bulimia nervosa (BN), binge eating disorder (BED)**.

	AN	BN	BED
BMI	<17.5 kg/m^2^	>17.5 kg/m^2^; <25 kg/m^2^	>17.5 kg/m^2^
Lifetime prevalence	1.9–2.6% (3)	0.5–1.5% (4)	2–3.5% (4)
DSM-5	Distorted body image, excessive dieting	Recurrent episodes of binge eating followed by inappropriate purging behaviors (self-induced vomiting)	Recurrent episodes of eating significantly more food in a short period of time than most people would eat under similar circumstances, feelings of lack of control
Personality traits	Anxiety, fear to gain weight, avoidance, perfectionist, poor self-esteem, compulsivity dysmorphophobia	Anxiety, avoidance, poor interoceptive awareness, ineffectiveness, self-directedness, stress reactivity, perfectionism	Anxiety, poor self esteem, harm avoidance, impulsivity
Comorbidities	Anxiety, depression, TOC, addiction, phobia	Anxiety, depression, TOC, addiction, phobia (obesity)	Anxiety, depression, TOC, addiction, phobia, obesity

Anorexia is said to be restrictive if during the past 3 months the person has not engaged in recurrent bulimic crises or purging behavior (i.e., self-induced vomiting or abuse of laxatives, diuretics, or enemas). One might consider this restrictive AN (AN-R) subtype as an awful eating disorder as the chronic refusal to eat leads to severe and sometimes irreversible complications for the whole organism, until death. AN-R is considered as a multifactorial disease with a complex etiology. The dramatic physiological and psychological consequences on health generated by the low food intake might lead to central and/or peripheral reprograming that permits the organism to endure in a first step, this reduced energy supply. A better understanding of the different facets of this disease becomes an urgent necessity to find novel therapeutic approaches complementary to the classic psychological therapies.

The objectives of this review are first to present briefly the main pathophysiological alterations observed in AN-R patients, then to introduce the different animal models that are currently used or could be used to better apprehend the physiological, metabolic, and neurobiological dysfunctions associated with AN-R, and finally to discuss the potential contribution of these models for understanding the pathology.

## Physiological Alterations in Restrictive Anorexia Nervosa: From Neurobiology to Genetic Polymorphisms

The two faces of anorexia, physiological and psychological, which were first used to describe the disease, then were neglected by the psychiatrists and psychologists for years are now more and more widely accepted by numerous clinicians and practicians to interfere.

### Physiopathological alterations

The recent DSM-5 (2013) suggests diagnosing AN-R by three major criteria. The first criterion is a severe and persistent restriction of energy intake leading to significantly low body weight in context of what is minimally expected for age, sex, developmental trajectory, and physical health. The gradual loss of weight can reach more than 50% of the initial body weight. The second criterion is the intense fear of gaining weight or of becoming fat. The third criterion is a disturbance in the way AN patients experience their body weight or shape (dysmorphophobia), associated with persistent lack of recognition of the seriousness of the current low body weight. Another important criterion, amenorrhea or the absence of at least three menstrual cycles, was removed in the DSM-5. This criterion was deleted since it cannot be applied to patients of different age and gender. Moreover, some data describe individuals who exhibit all other symptoms and signs of AN, but still report some menstrual activity (5, 6).

Anorexia nervosa has one of the highest mortality rates of all psychiatric diseases (7, 8). In a 21-year follow-up study, Löwe et al. (9) showed that 16% of AN patients deceased due to consequences of the illness. Among them, about 50% died because of somatic complications and the other 50% committed suicide. In fact, the course of AN is extremely variable, with approximately 50–60% of individuals with AN that recover, 20–30% that partially recover, and 10–20% that remain chronically ill (9, 10). Among the different clinical studies conducted on AN patients, low ionic plasma concentrations, symptomatic hypoglycemia, and anemia are often associated with lymphopenia that can generate opportunistic infections or hepatic cytolysis in some cases. However, contradictory results were published concerning essential amino acid levels in plasma of AN patients and healthy controls (11–13). Modifications in essential metabolites might be related to the generalized amyotrophy often described in AN patients. Moreover, increase in the metabolic hormone levels (like ghrelin or cortisol) is often observed, and the endocrine function of adipose tissue is modified resulting in increased circulating levels of adiponectin and decreased concentrations of leptin (14, 15). Usually, AN-R patients also showed a nutritionally acquired hepatic resistance to GH with decreased production of IGF-1 and increased GH levels. Such increase is due to (i) a reduction of IGF-1 feedback on pituitary and hypothalamus GH secretion and (ii) high levels of ghrelin, a GH secretagogue (16). Additionally, osteoporosis, another main complication of AN affecting 20–50% of cases, has been observed and is often irreversible (17, 18). Behavioral changes like physical (or intellectual) hyperactivity observed in 31–80% of the cases might also be associated with AN (19). Finally, disordered fluid intake is currently associated with AN-R, 54% of patients drinking excessively, and 28% drinking restrictively (20). This leads to relatively frequent renal complications (21).

### Neurobiological alterations

Anorexia nervosa is often associated with psychiatric comorbidities like depression, anxiety, obsessive–compulsive or personality disorders, and drug abuse (22). It becomes more and more accepted that AN-R resembles an addictive behavior disorder linked to food deprivation, weight loss, or physical activity. In fact, neuroimaging studies have first pointed out morphological changes affecting gray and white matters (23). The systematic review of Phillipou et al. (24) summarizes a number of brain differences, which are reported in AN patients. The neural profile of AN corresponds to a predominant imbalance between the reward (meso-cortico-limbic system) and inhibition (prefrontal cortex) systems of the brain. Recent data of Kullmann et al. (25) suggest that AN patients showed a reduced connectivity in the brain areas involved in the cognitive control and an increased connectivity in regions important for salience processing. The demonstrated altered integrity of the inferior frontal cortex might contribute to the physical hyperactivity developed by AN patients due to its role in the general behavioral inhibition like motor response. Furthermore, dysfunction of the central monoaminergic systems has been related. The review of Bari and Robbins (26) describes the implication of these systems as pathological neural substrates of diseases. They underline that prefrontal noradrenergic neurotransmission is involved in the inhibition of an already initiated response whereas dopaminergic system appears to modulate motor readiness for both inhibition/activation and reward, respectively at the level of the dorsal and ventral striatum. Dopamine has been associated with the expression of an appetitive reward system (27), and probably works in mutual opponency with a system that signals the prediction of punishment instead of reward. Serotonin neuromodulation might contribute to the more affective part of the inhibition behavior and/or the wanting behavior. Serotonin has a critical role in the adaptation of animals to aversive events, in the inhibition of appetite, and in anxious and obsessive behaviors, as well as in depression. Furthermore, harm avoidance is a temperament trait highly observed in AN patients (28), that reflects inhibition and anxiety and involves both dopamine and serotonin (5-HT) neurotransmission (29). AN patients show decreased dopaminergic metabolite levels in the cerebro-spinal fluid as well as increased dopaminergic D2/D3 receptor density (30, 31). Similarly, levels of serotonin markers like blood serotonin contents, plasma tryptophan are lower in AN patients compared to non-eating disordered subjects (32). Brain imaging studies using serotonin-specific radioligands have consistently shown 5-HT1A receptor binding is increased in cortical and limbic structures in ill and recovered AN patients (33, 34), whereas 5-HT2A receptor binding remains normal in ill patients (33). 5-HT transporter activity is also increased in recovery AN patients (35). The basal hyperfunctioning of the serotonergic pathway described in these various studies may be related not only to alteration in the reward process of food intake but also to anxiety, behavioral inhibition, and body image distortions (29, 36, 37).

Finally, one might also consider the involvement of the endocannabinoid neurotransmission in the neurobiological changes observed in AN patients. As reviewed by Monteleone and Maj (38) in a positron emission tomography study, AN patients showed a dysregulated endocannabinoid tone with enhanced plasma anandamide (AEA) levels and an increased number of cannabinoid type 1 receptors (CB1) in the insula and inferior frontal and temporal cortex of underweight AN patients. These data underline or suggest that altered food intake in AN patients may be a consequence of aberrant reward processing combined with an exaggerated cognitive control [see review in Ref. (39)]. Consequently, the current psychopharmacologic strategy in the treatment of AN uses typical and atypical antipsychotics, tetrahydrocannabinol, anticonvulsants, antidepressants, which modulate the synaptic signals of these neuromediators, but which have been illusive for decades [see Ref. (40)]. Thus, dissecting the mechanisms of action of the different neuropeptides/neurotransmitters involved in the regulation of food intake, as well as in the motivational aspects of feeding, becomes a necessity to open new perspectives for an efficient therapy of this disease complementary to the psychological approaches.

### Genetics

As clearly summarized by Scherag et al. (41), formal genetic studies suggested a substantial genetic influence in eating disorders and particularly in AN. The possible involvement of genetic components was strengthened by several twin and family studies concluding that AN presents genetic etiological components for 33–84% of the patients (42–45). Beside this, genome-wide linkage screens have been performed in order to identify unknown genes involved in AN. In the following paragraphs, presented data without reference to publication where cited in Scherag et al. (41).

Investigation on the genes directly involved in the regulation of feeding and energy expenditure was performed. The *leptinergic–melanocortinergic* system includes several key factors of the regulation of food intake and body weight. Surprisingly, despite the anorexigenic role of the leptin hormone, critically involved in the regulation of energy balance and adaptation of organism to semi-starvation, mutation analysis of the leptin gene and of the leptin receptor gene did not show any association with AN (46, 47). Agouti related peptide (AgRP), an orexigenic peptide, acts downstream of leptin through inhibition of central melanocortin receptors (MC receptors). Several studies concluded that the Ala67Thr AgRP polymorphism is significantly associated with AN. However, the involvement of this polymorphism in AN patients remains to be determined. This mutation would cause a lower inhibition the MC4R, a decrease in food intake, and would increase the risk of developing anorexia (48, 49). Brain-derived neutrophic factor (BDNF) is indirectly involved in the negative control of food intake. Low plasma levels of BDNF were determined in acute patients with AN. Several studies found that variants of BDNF and BDNF receptors (TrkB) are associated with AN. Moreover, AN patients often display high plasma levels of *adiponectin*, an adipocyte hormone known to play a role in the regulation of food intake and energy expenditure. Recently, a German study showed that several single nucleotide polymorphisms within the adiponectin (AdipoQ) locus were associated with adiponectin serum levels or eating behavior (50), but there is no convincing published study on the linkage between adiponectin gene polymorphism and AN.

Among the neurotransmitters suspected, genes involved in the *serotonergic* and *dopaminergic* systems have been pointed out. An overexpression of serotonin was suggested in AN. An association was shown with AN for serotonin transporter, serotonin receptors, and tryptophan hydroxylase 2 expressions. Moreover, positive but non-significant associations were also observed for dopamine D2 and D4 receptors and catechol-O-methyltransferase genes. The *norepinephrine* system was also investigated as low norepinephrine serum levels were always measured in recovery AN patients. Variants of the norepinephrine transporter gene that could lead to a lower norepinephrine reuptake have been associated with AN.

The *endocannabinoid system* is particularly involved in the regulation of appetite, food intake, and energy balance. Cannabinoids stimulate food intake through activation CB1. A study on 52 families showed that an allele of CB1 gene was more often transmitted in the restricted AN group. Moreover, in the Japanese population, Ando et al. (51) showed an association of a polymorphism of fatty acid amide hydrolase, which role is to inhibit the activity of the main CB1 ligand (N-arachidonoyl-ethanolamide), with AN.

As a first general comment on these data, it is to note that for several genes there is no evidence to suggest that any of the polymorphisms identified has a functional consequence on the biological activity or expression of the resulting protein. This may lead us to ponder these data when we try to establish linkages between polymorphisms and physiology or etiology. A second conclusion is that most of the polymorphisms that were shown to be associated with AN are related to the central nervous system, and particularly factors involved in the regulation of energy balance.

## Inputs of Animal Models of AN

Development of appropriate animal model of AN appears to be something difficult given the complex etiology. Although psychological factors play a pivotal role in the development of AN, a better understanding of the biological basis of this eating disorder can help to improve current treatments additional to therapies currently used by psychologists and psychiatrists. However, due to obvious ethic reasons, all the aspects of AN remain difficult to assess rendering necessary to develop relevant animal models. Thus, in rodents, different genetic and environmental models have been developed with varying degrees of success.

### Genetic models

Two categories of genetic models are commonly used: models presenting spontaneous mutations and genetically engineered models that can be constitutive or conditional.

#### Spontaneous Mutations

##### Anx/anx mice

This model has been extensively studied and described (52). The mutant mice *anx/anx* emerged spontaneously in the Jackson Laboratories (Bar Harbor, USA) in 1976. These mice are characterized by an emaciated appearance, a reduction in food intake, and early death 3–5 weeks after the birth (53). Moreover, serotonergic hyperinnervation and decrease in the striatal dopamine concentration and its metabolites may contribute to alterations in the locomotor and reward systems (54, 55). The *anx/anx* phenotype is associated with an approximative 50% downregulation of the gene *Ndufaf1* in the hypothalamus. It encodes a protein required for assembly of mitochondrial complex I (56). These mice exhibited several deviations in the hypothalamic neurotransmitter and neuropeptidergic systems involved in the regulation of food intake and energy metabolism, with a down-regulation of anorexigenic peptides POMC and CART and variations in the expression of the orexigenic NPY and AgRP peptides in the arcuate nucleus (54, 57–59). The reduction of the leptin peak, usually observed around postnatal day 8, could alter the arcuate neuronal development (60). These data are associated with mitochondrial dysfunction and neurodegeneration/neuroinflammation processes (52, 56, 61, 62). All these data suggest that this natural genetic model of anorexia represents an excellent model of anorexia–cachexia syndrome characterized by an inflammatory response that might be useful to dissect mechanisms that lead to physiological dysfunctions observed in AN. Here, the main limitations of this genetic anorexia model are: (i) the premature death of the mice before reaching puberty and (ii) effects observed on both male and female mice.

##### Lou/C rats

Lou/C rat is a rat substrain obtained from a Wistar rat selection at the Louvain University (Belgium). Lou/C rats are mainly characterized by a long life span until 35 months in male and 40 months in female (63, 64). These rats present the particularity to be resistant to diet-induced obesity and age-induced obesity since they exhibited a spontaneous food restriction, by eating fewer calories per day than Wistar rats in standard chow diet (63). The decreased food intake level is associated with a lower body weight (65, 66) itself associated with high energy expenditure and high sympathetic tone in the white and brown adipose tissues (67). Interestingly, Lou/C rats develop also an osteoporosis related to age associated with increased bone marrow adiposity (68). Lou/C rats mimic leptin, insulin, ghrelin, GH, and IGF-1 alterations observed in AN patients (66, 69–71). At central level, Lou/C rats present an upregulation of the hypothalamic AgRP, NPY, and orexin mRNA, and a down-regulation of leptin and ghrelin receptors in the arcuate and ventromedial hypothalamic nuclei (70).

Even if this rat strain presents various common alterations observed in AN patients, it is a more suitable model of healthy aging (64, 72).

#### Genetically Engineered Mice

Beside spontaneous mutation models, various genetically engineered models have been developed. In humans, genomic association studies have shown that various gene polymorphisms seem particularly linked to AN (see “Genetics”). In view of these data, we have summarized results from studies on animal models with modified genes encoding molecules involved in neuropeptidergic circuits and monoaminergic systems. For a more complete overview, animal models based on genetic alterations of peripheral factors are also presented (Table 2). It is noteworthy that the interest of all these models is discussed independently of the purpose of the original studies and thus of their intrinsic interest.

**Table 2:** **Presentation of the most pertinent model to decipher subtle peripheral and central mechanisms that might be involved in anorexia nervosa**.

Gene	Main peptide functions	Gene alteration mimicking AN alteration	Main induced alterations	Reference	Comments related to AN alterations
Leptin or Leptin receptor	Regulation of energy balance, food intake	Deficiency	Hyperphagia, obesity, diabetes	(73–76)	No mimicking the main AN alterations, models of obesity and diabetes
PYY	Anorexigenic in response to food intake	Overexpression	Reduced food intake after short fasting, normal body weight, and energy expenditure	(77, 78)	No mimicking the main alterations
Ghrelin	Orexigenic, energy balance	Overexpression	Increased food intake but normal body weight	(79)	No mimicking the main alterations
Goat and ghrelin	Activation of ghrelin (acylation)	Overexpression	Decreased energy expenditure but normal food intake and body weight	(80)	No mimicking the main alterations
Pancreatic polypeptide	Regulation of gastric emptying, …	Overexpression	Modest decrease of food intake and body weight	(81)	Slightly mimicking food intake and body weight alterations
Cholecystokinin	Satiation peptide	Deficiency	Low lipid absorption, normal food intake, and body weight	(82, 83)	No mimicking the main alterations
Neuropeptide Y	Orexigenic, decrease in energy expenditure and anxiety	Deficiency	Normal food intake and body weight	(84)	No mimicking the main alterations
Neuropeptide Y	Orexigenic, decrease in energy expenditure and anxiety	Destruction of NPY neurons in adults	Decreased food intake and body weight	(85)	Mimicking the voluntary food restriction and body weight decrease
Y2/Y4 receptor	Orexigenic, decrease in energy expenditure and anxiety	Deficiency	Normal food intake, lower body weight, higher activity, and energy expenditure; lower anxiety- and depression-related behavior for Y4	(86, 87)	Mimicking the body weight decrease
Agouti-related peptide	Orexigenic, decrease in energy expenditure	Destruction of AgRP neurons in adults	Decreased food intake and body weight	(85)	Mimicking the voluntary food restriction and body weight decrease
Melanin-concentrating hormone (MCH)	Orexigenic, regulation of physical activity	Deficiency	Decreased food intake and body weight, increased activity	(88, 89)	Mimicking voluntary food restriction, body weight decrease, and high activity
Cannabinoid type 1 receptor (CB1)	Orexigenic, regulation of energy expenditure	Deficiency in hypothalamus of adult	Normal food intake but lower body weight gain associated with a greater energy expenditure	(90, 91)	Mimicking the low body weight
5-HT4	Serotonin receptor	Deficiency	Voluntary food restriction following restraint stress; reduction of novelty-induced exploratory activity	(92)	Mimicking the voluntary food restriction
5-HT4		Knockdown in Accumbens nuclei	Increase food intake in fed mice	(93)	No mimicking the main alterations
5-HT1B	Serotonin receptor	Deficiency	Decrease food intake	(94)	Mimicking the voluntary food restriction
5-HT1A	Serotonin receptor	Deficiency or chronic agonist treatment	Decrease food intake	(95)	Mimicking the voluntary food restriction
Tyrosine hydroxylase	Production of dopamine	Deficiency in dopaminergic neurons	Strong hypophagia and hypoactivity; need of dopamine treatment to survive	(96)	Mimicking the voluntary food restriction but not the hyperactivity tendency
BDNF	Neurotrophin factor which stimulates growth and differenciation of neurons	No model of overexpression	Inhibit food intake	(97, 98)	No mimicking the main alterations
M3 receptor	Acetylcholine receptor or muscarinic receptor	Deficiency	Decrease food intake, lower body weight; hypoactivity	(99)	Mimicking voluntary food restriction and some endocrine alterations
CRH	Stress reaction	Deficiency	Decrease food intake, lower body weight	(100)	Mimicking the voluntary food restriction and low body weight
CRH		Central overexpression	Increase food and water intake; increase body temperature and heart rate	(101)	No mimicking the main alterations

##### Peripheral factors: hormones involved in the regulation of the energy metabolism

As a consequence of *leptin* anorexigenic function, leptin-deficient (*ob/ob*) or leptin receptor-deficient (*db/db*) mice display a phenotype of hyperphagia and obesity [see reviews in Ref. (76, 102)]. Even if the plasma levels of leptin are low in AN, these genetic models did not mimic pathology alterations. Mice overexpressing the *ghrelin* peptide in their stomach show higher plasma levels of bioactive (acyl) and total (acyl and non-acyl) ghrelin. They display a slight increase in food intake but not in body weight (79). To increase acyl-ghrelin plasma levels, it might be necessary to also increase the expression of GOAT (ghrelin O-acyltransferase), enzyme involved in the ghrelin acylation. Contradictory results were obtained for GOAT expression levels in stomach after 12–36 h of fasting, whereas chronic and severe food restrictions (21 days, 70% restriction) increase GOAT expression in rat (103). Mice overexpressing GOAT display higher concentrations of acyl-ghrelin without any changes in body weight or food intake (80). Thus, engineering genetic alterations of the ghrelin system in mice did not succeed in mimicking AN alterations despite the essential role of this hormone in the maintenance of glucose homeostasis on food restriction condition (104–106). The anorexigenic peptide *PYY* is physiologically released in response to food intake and its plasma levels increased in patients with AN. Mice overexpressing PYY display normal weight gain and food intake (77). These observations could suggest that this model should be excluded from the list of AN models, but a recent study (78) showed that when PYY overexpression begins in adult mice, it induces a reduced food intake after 24-h fasting. However, these mice display no significant difference of body weight or energy expenditure when compared to wild type mice. The *pancreatic polypeptide* (PP) produced in pancreas after food intake inhibits gastric emptying, and contributes to the important satiety effect of cholecystokinin (CCK). Baseline PP concentrations were similar between AN patients and healthy controls (107) or higher in AN patients (108), but these concentrations increased much more in AN patients than in controls after a meal test (107, 108). Transgenic mice over-expressing PP display a slightly lowered body weight associated with a modest reduction of food intake (81). *CCK* is a gut hormone stimulated by fatty meals and inducing satiety. It is also involved in the control of gastrointestinal motility and in anxiety behaviors. The response of CCK to a meal test was four-fold lower in AN patients than in healthy control group (107). Interestingly, CCK deficient mice display a normal food intake and a normal body weight when fed a basal diet (82, 83). Thus, once again, this model does not mimic the main alterations observed in patients with AN.

##### Neuropeptidergic systems

Modifications in the expression of neuropeptides permit to generate central alterations that might explain mechanisms giving rise to some of the symptoms described in AN patients.

In the arcuate hypothalamic nucleus, the two populations of orexigenic and anorexigenic neurons and their receptors, respectively the AgRP/NPY and αMSH/CART (α-melanocyte stimulating hormone/cocaine amphetamine related peptides) neurons, have been the focus of numerous studies in an attempt to better understand the finely tuned regulation of food intake. During fasting, NPY and AgRP gene expressions are up-regulated, and αMSH and CART gene expressions are down-regulated in hypothalamus. Moreover, various experiments suggest that NPY/AgRP inhibits directly the activity of αMSH neurons through a corelease of GABA, as well as an action on MC4R-bearing cells. Inactivation of genes encoding NPY, AgRP, or both has little effect on energy balance (109). Mice KO for *NPY* present significant changes neither in their body weight nor in their food intake, but become hyperphagic following food deprivation (110, 111). Surprsingly, mice KO for both Y2 and Y4 receptors exhibited a reduction in adiposity and an increase in lean mass, but without significant changes in food intake. Energy expenditure and physical activity were significantly increased in Y4-KO and particularly in Y2-KO/Y4-KO (87). Such models might be valuable to study the involvment of NPY and its receptors in the modulation of body composition and energy metabolism that are dramatically disturbed in AN. Contrary to the Y2 and Y4 receptors, the Y1-KO and Y5-KO mice develop the late-onset obesity with an increase in food intake and adiposity (112–114). This implies compensatory mechanism in feeding behavior in these KO mice and underlines the complexity of the NPY-food intake regulation system. Selective acute deletion of *AgRP* neurons in the adult mouse inhibits feeding and can lead to starvation not observed when the ablation is performed in neonatal mice before AgRP neurons are mature (85). Wu et al. (115) show in Ay/a mice no discernable effect on the anorexia phenotype caused by AgRP neuron ablation, suggesting that excessive activation of the melanocortin signaling is not responsible for starvation. Compensatory mechanisms may occur and hide the potential role of certain peptides (116, 117). Unfortunately, in these models, physiological data are rarely presented, their use are of interest to better understand the dialog existing between these populations of neurons by deciphering the involvement of their receptors in specific conditions. These approaches can highlight the main homeostatic pathway disturbed in AN.

The lateral hypothalamus contains *MCH* (melanin concentrating hormone) orexigenic neurons, described to be essential in the control of food intake and physical activity (88). In his review, Macneil (118) points out the various mouse models where disruption of MCH signaling results in altered energy homeostasis. Indeed, targeted inactivation of the MCH gene in mice induces reduced body weight and leanness due to hypophagia associated with an increased metabolic rate, despite reduced amount of both leptin and arcuate nucleus proopiomelanocortin mRNA (88). KO MCH mice also display an increased running-wheel activity during dark period (89). In the Promch/ataxin-3 mouse, 60–70% of MCH-expressing neurons degenerate in the first few weeks of life. Thus, at the age of 7-week, mice developed reduced body weight due to hypophagia and increased energy expenditure, body length, fat mass, lean mass, and leptin levels (119). Similarly, the Mchr1^−/−^ mice were less susceptible to diet-induced obesity, and the leanness was a consequence of hyperactivity and altered metabolism. The manipulation of the MCH system remains one of the most interesting to reproduce many of the symptoms described in AN. The progressive degeneration of an orexigenic neuronal population induces a voluntary food restriction that impacts the overall physiology of the animal.

The lateral hypothalamic area also comprises another population of orexigenic neurons: the *orexin/hypocretin (Hcrt)* neurons which are implicated in various functions altered in AN. Indeed, in a neuron-ablated strategy, the orexin/ataxin-3 transgenic mice severely reduced the formation of food anticipatory activity (FAA) under food restriction conditions (120). Furthermore, in a recent study, Ramanathan and Siegel (121) report gender differences in Hcrt KO mice. Hcrt KO females had increased body weight associated with increases in various components of the body composition, despite a decreased food and water intake not observed so drastically in the males. This promising model remains complex to interpret in the case of AN, because of the multiple roles in which orexin is involved.

Among the other neuropeptidergic systems involved in AN, the *cannabinoid* system must be pointed out. Mice invalidated for CB1 in hypothalamus showed a significant weight loss associated with greater energy expenditure despite a normocaloric food intake in standard diet (90). The mechanisms involved in such adaptations need to be more investigated since pharmacological manipulation of the endocannabinoid system is currently discussed as potential strategy for the treatment of anxiety disorders, depression, and AN (122, 123). Similarly, the *opioïd* system is known to play a role in the control of homeostatic and hedonic pathways. Thus, mice knockout for the opioid receptors like the μ-receptor display no significant difference in body weight, food intake, locomotor activity, or dark respiratory quotient when fed with regular chow diet compared to wild type mice, but they are resistant to diet-induced obesity and display more important weight loss during food deprivation (124–126). They also show a decrease in food motivation as demonstrated in an operant paradigm for chow diet or sucrose pellets, and a reduction of FAA in a daily scheduled food access compared to wild type mice (127, 128). These models might be of interest more specially to dissect the complex mechanisms that regulate the non-homeostatic aspects of the feeding in AN patients.

##### Neurotransmitters: dopamine and serotonin

As mentioned above, in AN patients, neuroimaging studies as well as dosages in the cerebro-spinal fluid report alterations in the serotonergic and dopaminergic systems.

Concerning the *serotonergic* system, pharmacological treatments that increase serotonin disponibility lower consumption of food in humans and rodents (129, 130). The model of mice genetically modified for 5-HT4 receptors has been extensively studied as a model of anorexia (131). Briefly, these mice were characterized by a voluntary food restriction, only following restrained stress, and by an attenuation of novelty-induced exploratory activity (92). Conversely, the knockdown of 5-HT4 receptor in nucleus accumbens increases food intake only in fed mice (93). Likewise, mice lacking 5-HT1B receptor food restricted (20%, 3 days) eat less than the wild type mice when standard food ration is given. They also show an increased locomotion (94). Mice lacking 5-HT1A receptor or wild type mice chronically treated subcutaneously with a 5-HT1A receptor agonist display a decrease of their food intake (95). The interpretation of data obtained from manipulation of the serotonergic system is rendered difficult due to the large number of receptors and the various effects they have depending of their location at the synaptic level and in the brain. Thus, to better elucidate the role of serotonin in the feeding behavior, it is preferable to use conditioned deletion or the cre-lox technology to avoid large effects that might be more the result of compensatory mechanisms than a true action of the neurotransmitter.

Concerning the *dopaminergic* system, Szczypka et al. (96) used initially a gene-targeting strategy to inactivate specifically the tyrosine hydroxylase (TH) gene in dopaminergic neurons, sparing the production of dopamine as a precursor for adrenaline and noradrenaline. These mice, called “dopamine deficient mice,” became hypophagic and died from starvation at 34 days because they showed locomotor deficiencies. Routine treatment with l-DOPA restored a food intake similar to wild type mice. Using viral strategy (96, 132–134), the involvement of dorsal striatum and accumbens nucleus has been demonstrated in locomotion and motivation, respectively, underlining the importance of dopamine to execute behaviors necessary to seek and ingest properly food. In AN, dopamine deficiencies might contribute to alterations in the accomplishment of these behaviors. Moreover, motivation aspects of feeding are also under the influence of medial prefrontal cortex and amygdala as recently demonstrated and involved D1 and D2 receptors (135, 136). These recent data emphasize the complexity of the regulation of feeding motivation, complete brain imaging data obtained in humans in these brain regions, and point out the need of more targeted pharmacological treatments (137).

##### Other genes

Other genes are also studied in the case of AN and are potential targets involved in the maintenance and/or evolution of the disease, like BDNF (brain-derived neurotrophic factor), CRH (corticotropin-releasing hormone), the glutamate receptors, the muscarinic type receptors even if they are also involved in a variety of functions (138, 139) rendering difficult to dissect precisely their actual role in the regulation of hunger/feeding. BDNF, a neurotrophic factor, is also a central regulator of energy balance, since BDNF suppresses food intake by acting on hypothalamic neurons (97, 98). Unfortunately, to our knowledge, no studies on hypothalamic overexpression of BDNF and feeding behavior are described in the literature. Investigating the CRH system in the case of AN is rendered difficult, even if the link is obvious, since AN patients often present stress-related disorders like anxiety and depression. Among the genetically modified models, the CRH-KO mice model described by Jacobson (100), the mice fed with chow diet present a decreased food intake associated with a lowered body weight loss than mice fed with restricted protein diet. In the opposite, mice who overexpress central CRH display changes in autonomic variables, like increased body temperature and heart rate, as well as increased food and water consumption, when compared with wild type mice (101). Thus, as detailed along the review, the HPA axis plays a key role in the regulation of the homeostatic and non-homeostatic aspects of the AN altered feeding, but the precise role remains to be determined in this case due to numerous brain areas involved. Muscarinic receptors (M1 to M5) are involved in acetylcholine signaling and in various functions at peripheral and central level (140). The M3 receptor has been associated with alterations that are observed in AN. Indeed, M3 KO mice are hypoactive and display a voluntary food restriction associated with lower body weight compared to wild type mice. These transgenic mice also present lower fat deposits associated with reduction of plasma leptin and insulin concentrations. Moreover, M3 KO mice present an up-regulation of AgRP and down-regulation of POMC and MCH in hypothalamus compared to control (99). Due to the large distribution of these receptors in the CNS, targeted strategies of gene deletion must be chosen to assess precisely their involvement in the regulation of food intake (141).

##### Conclusion

The main results obtained on mouse models in which one gene expression was modified to follow the alteration of the corresponding protein levels described in AN patients was summarized on the Table 2. These lead us to mention that most of these models are more relevant for obesity or display no specific phenotype related to AN. Interestingly, this table points out that most of the alterations related to these genes induce phenotypes very different of the pathologic ones. This could be linked to the fact that alterations of factors in AN patients appear often to be opposite to the physiological and behavioral alterations obtained in these genetic models. As examples, the plasma levels of leptin and ghrelin, respectively, low and high in AN patients, might normally lead to an increase in food intake, which is not the case in the disease, reflecting a physiological adaptation that is not well-perceived at the central and/or peripheral levels.

Thus, even if these genetic models gave comprehensive informations about some mechanisms related to the processes regulating homeostatic and non-homeostatic regulation of food intake, these models are most often used on short term protocols and do not allow to follow the physiological and neurobiological evolutions of the phenotype while restrictive AN is usually a chronic disease. Furthermore, they focus on certain aspects of the disease such as hypophagia, hyperactivity, or motivational disturbances without taking into account a general view of the whole body functioning. To circumvent these drawbacks, the use of “environmental” model allows us to reconsider some of these aspects.

### Environmental models

Various environmental animal models have been proposed to mimic various symptoms of AN. These models are usually based on qualitative or quantitative modifications in the pattern of distribution of the meal, including period of quantitative food restriction or limited time of food access as well as exposure to chronic or acute stress.

#### Animal Model Based on One Inducing Factor

##### Dietary restriction models

Various studies have focused on adaptations induced by dietary restriction to determine contribution of energy imbalance or nutriment deficiency in changes observed in AN patients. Some studies focused on life span, cancer prevalence, or metabolic syndrome have brought data useful for understanding AN-related alterations [see review in Ref. (142)]. Altogether, the different feeding paradigms lead to various but complementary results.

##### Food restriction (FR)

Most of the studies using chronic FR used mild restriction protocols. Restricted animals were fed usually 30–40% less than *ad libitum* control ones. However, it must be noted that in animal facilities, rodents are usually overfeed of about 30% compared to their physiological needs resulting in a significant weight gain over the time and leading to the use of overweight animals as reference (143). In FR protocols, body weight changes are age and gender dependent. Breeding weaned mice onto 30% FR lead to gain weight, even less rapidly than control ones (144). On the contrary, feeding adult mice (10 weeks of age) with 30% FR induces a loss of 20% of their body weight in 1 week (145). Thus, such FR models should be considered as valuable models of balanced feeding as shown by the induced longer lifespan (146).

In the quantitative food restriction models, the severity of the restriction generates various levels of weight loss associated with modifications of energy expenditure and respiratory quotient (145, 147–150). Indeed, long-term 30% FR in mice leads to a significant shift to carbohydrate metabolism during the meal (145). In rats, a 30% FR applied during 48 h or 14 days induced a significant body weight loss associated with decrease in plasma leptin concentrations, but only acute food deprivation leads to a decrease in glycemia and plasma insulin concentrations. At central level, both protocols induce up-regulation of hypothalamic AgRP and NPY mRNA associated with down-regulation of POMC mRNA (151). A 30% FR applied for 9 weeks in 3–week-old mice impacts bone mineral content more rapidly than when it is applied in older mice (9–14 weeks old) (144, 152, 153). Food restriction is associated with emotional impairments (154). C57Bl/6 mice subjected to a 20% caloric restriction for 8–12 days exhibit an anxiety-like behavior (155). Moreover, in 20–30% FR rats for 7–10 days, a decrease in dopamine levels in the nucleus accumbens occurs associated with an impairment of the expression of genes related to the dopamine (156). These alterations could be involved in reward sensitivity and emotional and motivation related behaviors observed in AN patients.

##### Alternate feeding experiments

*Alternate feeding experiments* with animal fed 1 day every two days appeared to induce alterations close to that observed on 40% FR models. Mice under alternate feeding from 12 to 65 weeks of age displayed a 20% increase in their body weight while this increase reached 60% for control mice (157).

##### Severe food restriction

*Severe food restriction* studies (50–70% restriction) are much less common (158, 159). Because of their severity, these studies are often shorter while numerous changes need several weeks to develop (160, 161). However, in a 50% FR on a long term protocol, mice show a decrease in energy expenditure after a meal associated with a decrease in lipid oxidation (150). Severe FR on 5-week protocol induced emotional impairments on rats. They showed increased anxiety like behavior, decreased serotonin turnover in the hippocampus and hypothalamus, and a decreased expression of 5-HT reuptake transporter in the raphe nucleus (162). Alterations of dopamine and DOPAC levels in septum and hypothalamus are associated with conditioning fear and control in food intake (163–167). The dopaminergic signaling was also shown to be modified (168) in the mesolimbic circuitry, strongly involved in the modulation of the motivational aspects of the food intake. Altogether, these protocols mimic various AN symptoms such as body weight loss associated with alterations in reproductive function, metabolic, endocrine, and neuro-endocrine systems (Table 3). Moreover, these models bring very interesting informations about the potential mechanisms sustaining physiological alterations observed in AN, and due to chronic caloric restriction, but they do not take into account two other major components widely described in AN, namely stress and physical activity. Other models have been developed to determine the role and involvement of both of these factors.

**Table 3:** **Environmental models: main physiological and neurobiological changes observed in rodent models manipulated for one or several factors**.

	Inducing factors	Duration	Body weight and tissues	GH/IGF-1	Reproduction	Energy metabolism and appetite regulating hormones	Stress	Central impact (neuropeptides/neurotransmitters)	Key references
**Restrictive anorexia nervosa**	Not well known	Months to years	20–25% under normal weight (↘fat mass); osteoporosis	GH resistance (↗GH ↘IGF-1); ↗→SRIF in CSF; ↗↘SRIF in blood	Amenorrhea; ↘LH, FSH, E_2_	↘ Energy expenditure; ↘Leptin; ↘Insulin; ↗Ghrelin (acyl- and desacyl-ghrelin); ↗ adiponectin; ↘Glycemia	Anxiety-related behaviors and mood disorders; ↗Cortisol; ↘ACTH; →CRH	Morphological alteration of white and gray matter; ↗AgRP↗NPY; →αMSH in blood; ↘Dopamine metabolites in CSF, ↘D2/D3 density; ↘Serotonin markers	(30, 31, 169, 170) (review), (14, 18, 38) (review), (32)
**Animal models** Mild food restriction	30–40% food restriction	Months to a year	0–20% of weight loss (↘→lean mass, ↘fat and bone masses)	↘ GH; ↘IGF-1; →GHRH	→ GnRH	↘Energy expenditure; ↘Leptin, insulin; ↘→Ghrelin total, ↘Desacyl-ghrelin; →Adiponectin; ↘→Glycemia	Anxiety-like behavior; →ACTH; ↗Corticosterone	↗AgRP↗NPY; ↘POMC; ↘Dopamine and DOPAC in septum; ↗DOPAC/dopamine ratio in hypothalamus	(145, 151, 152, 155, 164, 171–173)
Severe food restriction	50–70% food restriction	24 h to 60 days	Until 20% of weight loss (↘lean, fat masses,↘bone mass)	↘GH; ↘IGF-1; ↗FGF-21	Stop estrus cycle; ↘LH, ↘FSH	↘Leptin, insulin; ↗Ghrelin (acyl- and desacyl-ghrelin); ↘Glycemia (15 days); ↗Free fatty acids; ↘Ketone bodies; →Triglycerides; ↘Energy expenditure	↗Corticosterone	↗AgRP↗NPY; ↘POMC; ↘Dopamine and DOPAC in septum; ↘DOPAC/dopamine ratio in hypothalamus	(150, 156, 164, 171, 174, 175)
Time-restricted feeding	6–1 h food access/day	Until 16 weeks	Lower body weight gain than control to 25% of weigh loss	?	?	↘Insulin; ↘Glycemia; ↘Triglycerides	↗→Corticosterone; →CRH; →ACTH	↘Anxiety-like behavior; ↘Serotonin in hypothalamus; Circadian clock disturbances	(176–179) (review)
Low fat and fat-free	Reduced fat intake	Two generations	20% of weight loss	?	Disruption of reproductive function	↘→Energy expenditure	?	↗AgRP↗NPY; ↗Dopamine signaling; ↘D2 binding, 5HT2A binding in frontal cortex	(180–185)
Low carbohydrate	Reduced carbohydrate intake	4 weeks	No modification or increase according food composition	↗GH; ↘GH receptor in liver; ↘IGF-1; ↘SRIF	?	↘Insulin fasted; →Ghrelin total, Acyl-ghrelin; ↘Glycemia fasted	?	?	(171, 186)
Low essential amino acids/protein	Reduced essential amino acid protein intake	2 days to 6 weeks	Until 30% under control weight	↘IGF-1; ↗SRIF	Stop estrus cycle	↘Insulin; ↗Ghrelin (acyl- and desacyl-ghrelin); ↘Glycemia; ↘Triglycerides	?	No anxiety and depression-like behaviors; ↘Serotonin turnover in brainstem, hippocampus, prefrontal cortex; involvement of anterior piriform cortex in aversion observed	(187–190) (review)
Dehydration-induced anorexia	Hyperosmolar drink (2.5% NaCl)	4 days to 2 weeks	Until 69% of the body weight of controls	?	?	↘Leptin, insulin; ↘TSH, T_3_	↗Corticosterone; ↘CRH, CRH-R2	↗NPY; ↘POMC; ↗ORX; ↗TRH	(191–193)
Restraint stress and immobilization	Slight contention 30 min to 6 h/day	1–42 days	15% of weight loss (↘lean, fat masses, ↘bone mass)	↘→GH	↘LH; ↘Testosterone	↗Energy expenditure	↗Corticosterone; ↗→CRH; ↗→CRH-R1	↗NPY, ↗AgRP; ↗POMC; ↗MCH, ↗ORX	(194–199)
Cold exposure	Exposure to 4 to −15°C	24 h to 4 weeks	Low body weight loss (↘lean, fat masses)	?	?	↘Leptin insulin; ↗Glycemia; ↗Free fatty acids	↗Corticosterone	↗MCH; ↗TRH	(105, 200–202)
Chronic mild stress	Random stress	5 days to 8 weeks	No or low body weight loss (↘ fat mass)	?	?	↘Leptin, insulin	↗CRH	↘NPY	(203–205)
Social stress	Group of rodent with an organization into a hierarchy	2 weeks and recovery phase	10–15% of body weight loss (↘fat mass)	?	?	↘Leptin, insulin	↗Corticosterone; ↗ACTH; ↗CRH	↗NPY; ↘Preproenkephalin in nucleus accumbens; ↗D2 binding in striatum	(206–208) (review), (209)
Activity-based anorexia (ABA)	Voluntary physical activity and time-restricted feeding	3–14 days	Stopped over 20–25% of weight loss (↘lean and fat masses)	?	Stop estrus cycle	↘Leptin, ↘insulin; ↗Ghrelin (acyl- and desacyl-ghrelin); ↘Glycemia; ↘Free fatty acids	↗Corticosterone; ↗Adrenal gland mass; →CRH	↗AgRP, ↗NPY; ↘POMC; ↘CART; ↗Dopamine during feeding in accumbens nuclei; ↘Serotonin in accumbens nuclei	(49, 210–215) (review), (216) (review)
Food restriction and wheel (FRW)	Voluntary activity and food restriction	15–55 days	18–22% of weight loss (↘lean, fat, and bone masses)	?	Stop estrus cycle	↘Leptin; ↗Ghrelin (acyl- and desacyl-ghrelin); ↘Glycemia (15 days); ↗Free fatty acids; ↘Ketone bodies; →Triglycerides; ↘Energy expenditure	↗Corticosterone (15 days) = Corticosterone (55 days)	?	(150)
Separation-based anorexia (SBA)	Stress related to separation and time-restricted feeding	Until 10 weeks and recovery phase	Until 28% of weight loss (↘lean and fat, ↘bone masses)	↗GH; ↘IGF-1	Stop estrus cycle	↘Leptin; →Glycemia	↘ACTH; ↗Glucocorticoïd	↗MHPG/norepinephrine in hippocampus; ↘Dopamine in hippocampus	(161, 217, 218)

*↗ increase; ↘decrease; →no changes of expression or concentration according to the compartment studied; ? not well-documented*.

*5HT2A, serotonin receptor 2A; ACTH, adrenocorticotropic hormone; AgRP, agouti related peptide; CART, cocaine and amphetamine regulated transcript; CRH, corticotropin-releasing hormone; CRH-R, corticotropin-releasing hormone-receptor; D2 receptor, dopamine receptor 2; DOPAC, 3,4-dihydroxyphenylacetic acid; E2, estradiol; FSH, follicle stimulating hormone; GH, growth hormone; GHRH, growth hormone-releasing hormone; IGF-1, insulin-like growth factor 1; LH, luteinizing hormone; MCH, melanin-concentrating hormone; NPY, neuropeptide Y; ORX, orexin; POMC, pro-opiomelanocortin; SRIF, somatostatin; TRH, thyrotropin-releasing hormone; αMSH, alpha-melanocyte-stimulating hormone*.

*The gray parts of the table point animal models induced by several factors*.

##### Time-restricted feeding (TR)

Time-restricted feeding consists in *ad libitum* energy intake, but within few hours each day. Recently, Rothschild et al. (179) wrote a comprehensive review on the links between TR and metabolic diseases in animal models and human. Sherman et al. (219) showed that a 3-h food access each day for 16 weeks induces a food intake 15% lower and a body weight increase a half lower in adult male mice compared to their *ad libitum* control mice. But in these experiments, restricted animal are fed during the light period. Longer durations of daily food access were also studied, but they had a lower impact on food intake and body weight. Most of the time-restricted studies demonstrated slight or no changes in body weight gain when compared to control group, but an improvement of markers of metabolic disease risks. They also pointed out the link between disruption of the molecular circadian clock and metabolic disorders even under high fat diet (219, 220). These models mimic neither severe food restriction nor body weight decrease described in AN. TR feeding also leads to a reduction in the anxiety-like behavior and alteration of the serotonin system of rats (176, 221). The authors suggest that the decrease in the essential amino acid tryptophan in the hypothalamus may be the consequence of plasma tryptophan decreases, and thus contribute to the decrease in the serotonin synthesis. The related hypothalamic variations are suggested to provoke a compensatory upregulation of postsynaptic 5-HT receptors to precipitate AN.

##### Low fat diet

Animal models based on low fat diet (4–5% of fat/g) could take into account the fact that patients with AN not only reduce their food intake, but also select their foods. But two main difficulties limit the use of these models to study AN. First, foods with 4% of fat are commonly used as low fat diet, even if this is the fat level suggested for standard rodent food, while 10% fat diets usually lead to overweight with time and age. Second, almost all studies focused on comparisons between high-fat and low-fat diet consequences or focused on the effects of low-fat diet on obese mice.

##### Fat-free diet

The first studies conducted on rats submitted to fat-free diet during from 60 days to 6 weeks display a decrease of body weight (80% compared to control), a lower growth with emaciation appearance associated with increase of water intake, no difference in food intake compared to control rats (180, 181). It was also described impairment of reproductive function in male and female rats (181, 222). Respiratory quotient measured in rats under fat-free diets (1 month) but submitted to carbohydrate access following 14 h of fasting evidenced a shift to lipid metabolism (182). Variations of plasma lipid induced by low-fat diet and fat-free diet are sensed by neurons of ventromedial hypothalamus (223–225). However, to our knowledge, only the study of Staszkiewicz et al. (185) showed an upregulation of AgRP and NPY expression in low-fat diet group. In parallel, a lower dopamine signaling is described in rats submitted during two generations of α-linolenic acid deficient diet compared to normal chow diet as well as a lower 5-HT2 binding was observed till in the frontal cortex, even if no significant difference was observed concerning body weight between groups (183, 184, 226, 227). Moreover, although these neurotransmitters are known to be related to anxiety- and depression-like behaviors, no behavioral test was conducted in these studies.

##### Low carbohydrate diet

Patients with AN also select food with low carbohydrate content in the aim to reduce their calorie intake. But in rats, low carbohydrate diets moderately impact body weight (171), and mice on a zero-carbohydrate diet significantly gain more weight than animals consuming standard chow, despite similar caloric intake. These zero-carbohydrate fed mice also exhibited metabolic disruptions, while low carbohydrate diets in humans induce greater weight loss than isocaloric food (228). These results do not lead to consider low carbohydrate diet fed mice as relevant models for AN. Finally, studies on high/low fat or high/low carbohydrate diets revealed great differences in the use of fat and carbohydrate between mice and humans.

##### Indispensable amino acid deficient diet (IAA)

Indispensable or essential amino acids are neither synthesized nor stored in organisms. In AN patients, one might consider that severe food restriction may alter the concentrations of plasma essential amino acids and might have drastic nutritional consequences (229). Several studies examining plasma amino acid levels display conflicting results in AN with higher, lower, or no significant differences compared to controls (11–13). However, a decrease in plasma tryptophan and a decrease in the tryptophan/large neutral amino acid ratio in acutely underweight AN patients are usually observed (32, 230–233). Thus, animal models based on essential amino acid restriction do not appear to be suitable models for AN. However, they could mimic some induced alterations, because essential amino acid restriction induces an adaptive behavior of food deprivation or because they are related to tryptophan. Various protocols have been developed using more commonly threonine, leucine, or valine deficient diets (189). Interpretation of changes observed should be taken with caution, since some alterations are related to energy deficit and others are related to the amino acid deficiency itself. In particular, valine deficient diet induced food restriction, a greater weight loss than for other IAA diets (approximatively 20% of their initial body weight), and increased of plasma acylghrelin and des-acylghrelin concentrations after 6 days of protocol (188). However, this valine deficient diet must be taken with caution since it leads to neurotoxicity not observed with an isoleucine deficient diet for example (189, 234–236). In another series of experiments using a combination of IAA deficient diets, Narita et al. (189) showed after 15 days of protocol, a decrease of glycemia, plasma triglycerides, leptin, insulin, and IGF-1 levels as well as a blockage of the estrous cycle in diestrus stage. Chronic tryptophan deficient diets (until 6 weeks) in rodents also lead to a progressive decrease of body weight (237–239). In contrast to acute deficient diet, no anxiety- or depressive-like behavior was observed in rodent despite a decrease in tryptophan concentration and serotonin turnover in brainstem, hippocampus, and prefrontal cortex (238–241). Indeed, no sucrose preference was observed in acute deficient rats while an increase of sucrose consumption was observed in mice after 5 weeks of tryptophan deficiency (239, 241). Unfortunately, to our knowledge, no studies determine the alterations of brain circuits regulating energy homeostasis. Rodents also develop different strategies to overcome the amino acid unbalance, including stopping the ingestion of food, change in the choice of food; they also develop a foraging behavior (to find complementary food); they establish an aversion with a learning phase and memorization of taste and smell to avoid the consumption of deficiency food in future (190, 242–244).

##### Dehydration-induced anorexia

As highlighted in part I, AN patients present relatively frequent osmoregulation impairment and renal complications due to their drink intake behaviors (21, 245, 246). Gutman and Krausz (247) pointed out a drastic decrease of food intake after acute subcutaneous injection of a hypertonic solution in rat. A “dehydration-induced anorexia” (DIA) model was developed by Watts (248). It consisted in a scheduled consumption of a hyperosmolar solution of NaCl (2.5%). This protocol has been tested for 4–14 days (192, 248). It provokes reduced food intake with a negative energy balance that is similar to those seen in pair-fed food-restricted animals: weight up to 69% under the body weight of control rodents, increased corticosterone, lowered leptin and insulin plasma levels (191). The food restriction is due a change in the pattern of food intake, a reduction of meal duration, and an inhibitory effect on gastric motility (249, 250). At the central level, DIA and pair-fed groups share up-regulation of NPY and down-regulation of POMC mRNA in the arcuate nucleus, and up-regulation of orexin mRNA in the lateral hypothalamic area only in pair-fed groups (191, 193). Beside, a down-regulation of CRH mRNA expression in the paraventricular nucleus and higher plasma corticosterone levels are observed only in DIA group (192). This model displays some common alterations also observed in AN. However, the drastic changes in osmolarity, which are not always observed in patients, might limit the use of DIA to decipher the central and peripheral mechanisms that can lead to chronic renal failure.

##### Stress models

A growing body of literature associated stress and anxiety as critical factors in the development of eating disorders like AN (251). Several animal models have been developed to evaluate mechanisms linking response to stressful events and alterations of food intake. In this section, we will not discuss data related to anorexia induced by the administration of lipopolysaccharides or endotoxemia. The most extensive studies concern restraint stress, cold exposure, or chronic mild stress (CMS).

##### Restraint stress

In rodents, limiting movements for a determined period (30 min to 6 h) every day generates a stress and a body weight decrease depending on the duration and type of immobilization (195). Indeed, animals are immobilized in a plastic tube or by attaching the four limbs to metal mounts with adhesive tape. Body weight loss up to 15% impacts both lean and fat masses, and is associated with a voluntary food restriction after an acute stress session (like 2 h), or when the stress is repeated (195, 252–254). Moreover, this low body weight is maintained even after a recovery period (199). Repetition of the restraint stress induces long lasting increased plasma corticosterone, ACTH and ghrelin concentrations, and decreased plasma leptin and insulin concentrations (255–258). In long duration experiments, bone physiology alterations are also observed (259). Rats exhibit an increase of energy expenditure and body temperature during the stress followed by return to control values (199, 260). At central level, noticable changes in the activation and/or expression of genes involved in the control of food intake are described. Acute restraint stress increases the number of activated neurons in several brain areas compared to controls, while repeated stress effects are lowered probably because of habituation (261–263). In these studies, modifications in the activation of the HPA axis are the most documented. Restraint stress protocols increase plasma corticosterone concentrations, which are associated with an increased activation and expression of CRH in the paraventricular nucleus (198, 264). However, such increases are not anymore observed when the stress is repeated (198, 199, 265). Considering the anorexigenic effects of intracerebroventricular injections of CRH, this peptide has been suspected to be responsible for the voluntary food restriction observed in this type of protocol (265). Both acute and repeated restraint stress in rats induce decreased number of neurons immunoreactive for Fos and AgRP in arcuate nucleus, while the number of neurons immunoreactive for Fos and MC4R increases in the lateral hypothalamic area but decreases in the arcuate nucleus on the long term (263). Such reduction in MC4R cell activation may signify a desensitization of feeding regulatory pathways in the arcuate nucleus after repeated stress exposure that may be indicative of a shift toward more orexigenic behaviors, as signals promoting feeding become more prominent. In another study where a 2-week chronic restraint stress is applied on mice, inhibition of food intake occurs until the end of the first week and is associated with also an up-regulation of POMC mRNA in the arcuate nucleus (258). The data obtained with NPY are less clear since acute restraint stress increases NPY mRNA expression in the arcuate nucleus. This expression is normal in the case of chronic stress (266). Thus, the relative balance between orexigenic and anorexigenic pathway activation appears to be dependent on whether the stress is acute or repeated. Finally, stress induces a very rapid degradation of GH (267) and thus a decrease in plasma GH concentrations (268, 269). The release of somatostatin, a major inhibitor of the GHRH release, increases in the median eminence level following acute restraint stress, and thus might be a major factor in this GH drop (270).

These results suggest that food intake may be increased or decreased as a consequence of stress, and may play a role in eating disorders from anorexia to binge-eating leading to obesity and other stress-associated metabolic disorders. Once again, this psychological stress impacts differentially the brain areas involved in the regulation of food intake rendering difficult to use such protocol to study precisely the mechanisms involved in AN.

##### Cold exposure

One hypothesis on the origins of hyperactivity often observed in AN is that it would be a form of thermoregulatory behavior. Studies on the effects of ambient temperature or heat treatment on AN patients displaying hyperactivity strengthen this hypothesis (271, 272). Cold exposure is a physiological stress used to determine mechanisms involved in control of body temperature. The protocol used temperature exposure from 4°C to -15°C and for a duration ranging from 24 h to 4 weeks. Usually, relatively low body weight loss is observed and is not always associated with a decrease in food intake (200–202, 273–276). This body weight loss is associated with a decrease in both lean and fat masses and an increase in brown adipose tissue mass (202, 277–279). Short term exposure (1–24 h) or long term exposure (8 days) to cold stress (at 4°C) increases blood glucose, plasma adrenaline, and corticosterone concentrations, and decreases plasma leptin and insulin concentrations (200, 276, 279, 280). Cold exposure also leads to increased glucose uptake by peripheral tissues associated with increased liver glycogen, lipolysis in white and brown adipose tissues, and concomitant to lipogenesis in these tissues (200, 279, 281). Activation of lipolysis in the different fat depots involves the sympathetic nervous system as suggested by an increased noradrenergic turnover (276, 277). Lower temperatures (under 0°C) during 2 weeks induced in mice, a more important body weight loss associated with higher food intake and lower body temperature (202). Cold exposure leads to activation of numerous brain areas involved in thermoregulation located in the hindbrain (282, 283), in the hypothalamus, and in the forebrain (280, 284). Cold exposure during 4 days (4°C) leads also to increase of MCH expression in the hypothalamus (201), suggesting the involvment of this neuropeptide directly or indirectly in such variations. However, the origin of these variations is unclear but they are probably due to the role of MCH in control of energy expenditure (201, 285).

##### Chronic mild stress

Depression is another sign classically observed in AN. The most valuable animal model of depression like behavior was developed by Willner et al. (203). This model called CMS consists to expose rodents to mild stress applied randomly and daily during 3 to 9 weeks. In this kind of protocol conducted on rodents, the body weight is slightly diminished but, a notable reduction of sucrose consumption, sign of anhedonia, is described (203, 204, 286, 287). The body weight loss concerns decreased subcutaneaous and visceral fat mass associated with decreased plasma leptin and insulin concentrations. However, these changes are not specific to CMS protocol because they are also observed in the “weight match” control group (205). At central level, CMS animals exhibit up-regulation of CRH in paraventricular nucleus while its expression is reduced in the “weight match” group (205). Other peptide expressions also are altered, with especially a down-regulation of NPY in the arcuate nucleus (204). The CMS is described to have anxiogenic effects through a stronger neuronal activation in various brain areas, as well as a decreased neurogenesis in the hippocampus (288). Recent reviews (289–291) pointed out a role of ghrelin in depression and anxiety, even if it is again still a subject of debate. Its receptor is present in structures known to be involved in mood disorders like hippocampus and amygdala. The model presents the advantages to mimic alterations of the stress axis and anhedonia for palatable food associated with a slight body weight loss. However, the complexity of the stress procedure and a rapid recovery limit the interest of CMS to mirror AN.

##### Social stress

Another kind of acute/chronic stress is related to rodent social interactions. The main models are based on social defeat stress and the visible burrow system (VBS). The social defeat stress was first used as a model of anxiety and depression (292). A rodent (intruder) is placed in the home cage of another rodent (resident). The interactions between the two animals are usually rapid and lead to aggressive behaviors, with a dominant and a subordinate. The defeat social stress leads to a markedly decrease of body weight in animal following 1 h session of stress (206). The repetition of this stress induces also a higher reactivity to an acute restraint stress with increased plasma corticosterone and ACTH concentrations, but with a normalization of values after stress (293). A decrease in locomotor activity is also observed associated with reduced social interaction in the presence of a non-aggressive rodent (206, 293). An increased nocturnal food intake is noticed and not observed in the case of VBS protocol (209, 293). The VBS protocol induces a more complex social defeat stress since it is based on the establishment of a hierarchy in a group of male rats leading to dominance hierarchies with offensive and defensive behaviors (294). At the end of the confrontation period, a dominant male rat (DOM) takes the ascendancy over other rats qualified like subordinate males (SUB). VBS protocol induces decrease of body weight associated with a decrease of food intake only in SUB male rats (208, 209, 295). The body weight reduction is associated only with a decrease in subcutaneous fat mass, whereas lean mass is unchanged and visceral fat mass is increased (208). The pattern of food intake is modified with a decrease of meal duration (209). SUB rats display also endocrine changes with a decrease of plasma leptin and insulin concentrations compared to DOM and control rats (296, 297). Studies related to alterations at the central level have mainly focused on the HPA axis, particularly affected in the SUB rats, with increased plasma corticosterone concentrations correlated with increased expression of CRH in the paraventricular nucleus and amygdala (208, 295, 296, 298). The chronically elevated corticosterone levels may create an orexigenic drive through upregulation of NPY and AgRP in the SUB rats as well as the loss of fat mass seen in both DOM and SUB, which indicates a negative energy balance, and may also create an orexigenic drive through similar mechanisms. Such observations are validated by the behavior observed in a recovery phase where the rodents become hyperphagic and increase drastically their fat mass (299). These observations render the model inadequate for studying the recovery period after food restriction even if the model generates transiently a food restriction during the protocol. It should be underlined that a noticable decrease of palatable food is observed as in the CMS protocol in the recovery phase (207, 208, 300). These changes have been attributed to alterations in dopamine transporter binding and dopamine receptor (D2) binding which are reduced or increased respectively in the striatum and accumbens nucleus in SUB group (207). The VBS protocol also leads to changes in the SUB serotonergic system in various brain areas involved in the modulation of stress (294, 301). This model is interesting to study the impact of chronic social stress on food intake and its homeostatic and non-homeostatic regulation. But the main drawbacks are: the absence in human of such notion of subordinate and dominant; the recovery period which shows a binge-eating behavior that is rare in recovered AN patients; the short term duration (around 15 days) excluding the development of long term alteration like osteoporosis. Finally, there is currently no or few data about the regulation of energy balance. The VBS model presents an important limit that reduces its use to study AN: it is applicable only on males.

#### Animal Model Based on Several Inducing Factors

##### Separation-based anorexia

Separation-based anorexia model is another model of chronic social stress not often used until now. This model is based on stress produced by a physical separation of mice belonging to the same group and associated with a food restriction or a time-restricted feeding (TR) (217). This study was initially conducted on Sabra female mice. Only few studies were published on this strain of mouse with high body weight. Food was provided during the light phase for 1 h a day. Control mice with the same feeding schedule lost 10% of their day 0 body weight within 18 days, and daily ate 2.84 g of food. Separated and time-restricted mice lost 28% of their initial body weight, and daily ate 2.33 g of food. In this group, 21% of mice died before reaching the targeted body weight loss of 33–35%. Separated and time-restricted mice ate 65% of the daily requirements and reach the same level of body weight loss than mice fed 40% of the daily requirements without being separated. These data suggested that separation of mice increases metabolic demands. This first study was followed by two studies on the same model and conducted by the same team. Both of them dealt with the effects of tyrosine treatments on central nervous system functions. Hao et al. (218) showed that SBA mice display an increase in 3-methoxy-4-hydroxyphenyglycol/norepinephrine ratio, an up-regulation of the cholinergic signaling, and a decrease in the dopamine concentration in hippocampus. In 2002, the effects of tyrosine treatments on HPA axis were studied on this model (302). This second central study pointed out a specific pattern of central alterations in SBA mice when compared to FR and active mice despite similar body weight loss. To allow studies on long term metabolic and central adaptations on a usual mouse strain, we recently adapted this model to C57Bl/6 young adult female mice. Food access was progressively reduced from 6 to 2 h a day within 2 weeks and then maintained at 2 h a day for up to 8 weeks (161). We have shown that this protocol induces significant weight loss with a reduction from 20 to 25% of initial body weight. Interestingly, the body weight loss observed in SBA group is not attributable to the timed food access as SBA mice eat only 10% less than *ad libitum* group. Moreover, such a difference in body weight is not observed in the TR group without separation. We suspect that this difference is partly due to rising energy costs both through the separation-induced stress and higher thermogenesis needs caused by the separation. Body weight loss is related to a decrease in lean mass and visceral and subcutaneous fat masses. In parallel, SBA mice present a blocking of their reproductive function and bone mass gain. Like in AN patients, various endocrine changes are observed. Thus, SBA mice display lower plasma leptin concentrations. Furthermore, disruption of the GH/IGF-1 associated with alteration in bone physiology was observed at 2 and 10 weeks. At metabolic level, protocol induces an up-regulation of several genes (UCP1, PGC1a, Prdm16) especially in the subcutaneous adipose tissue of SBA mice, suggesting the emergence of beige/brite adipocytes in this specific fat depot. Moreover, after 10 weeks of SBA, protocol mice were submitted to a 10-week recovery period with free food access in normal cage. During this recovery period, mice correct their various alterations including body weight, food intake, reproductive function, body composition, endocrine factors, and adipose tissue metabolism. However, SBA mice maintain low plasma leptin concentrations and low leptin expression in visceral fat tissue despite a full normalization of fat mass (161).

This long term model appears interesting as it mimics numerous central and peripheral alterations described or suggested in AN, and allows a recovery study. However, the increased energy expenditure related to chronic stress and high needs of thermogenesis does not match the decrease usually described in patients.

##### Activity models

In 1967, Routtenberg and Kuznesof developed a protocol, where rats isolated in a cage were allowed to have a timed food access, 1 h per day, combined to a voluntary activity. This model later named activity-based anorexia (ABA)/self-starvation/semistarvation-induced hyperactivity/food restriction-induced hyperactivity/wheel-induced feeding suppression model produces a rapid weight loss, close to 25% of their initial weight within days and food intake, physical hyperactivity, hypothermia, impaired estrous cycle in females, and increases in HPA axis activity (215, 303, 304). Moreover, rats eat less than inactive rats fed with the same schedule. This procedure led rapidly to a “self-starvation” or self-deprivation behavior resembling to that observed in restrictive AN patients and leading rapidly to the death of animals due to the voluntary privation of food (around 7 days). It is currently the most well-known animal model of anorexia (216, 305) and has been adapted to mice (306, 307). Recently, Lewis and Brett (308) reduced progressively the food access duration to maintain mice longer than 7 days. Following this new protocol, Jésus et al. (309) demonstrated alterations of intestinal permeability. In many aspects, all these models mimic numerous physiological alterations observed in AN. However, as specified in the review of Klenotich and Dulawa (310), the ABA paradigm is strongly dependent on the rodent strain, on age and gender (307, 311), on temperature [increasing the temperature to 32°C strongly reduces the ABA behavior, (312)], and on the time of the day the animals receive food. In fact, Boakes and Juraskova (313) and Boakes (210) demonstrated that the “self-starvation” observed in ABA rats might reflect both the reduced palatability of the dry chow for a dehydrated animal and satiety signals from a stomach full of water. Finally, in all these protocols, rodents were isolated in their cage to permit individual metabolic and physiological measures, but isolation creates a social stress adding on the physiological stress of food deprivation, rendering the protocol more drastic. Thus, all these studies present limitations that maintain a distance with AN. Recently, we have developed a modified ABA model on female mice, named here Food Restriction and Wheel (FRW) model that aims: (i) to prevent the social stress by using two mice per cage and (ii) to follow on the long term (up to 10 weeks) physiological alterations induced by a combination of physical activity and a food restriction of 50% (150). All of these activity models present metabolic, endocrine, and neurobiological alterations that might be the basis to study adequately some of physiological mechanisms altered in AN patients. Finally, they all exhibited a FAA, which occurs between 2 and 5 h before food intake distribution, and which is also described in AN patients (314).

The body weight loss observed both in ABA and FRW rodents is related to decrease of lean and subcutaneous/visceral fat masses after 7–14 days of protocol (150, 315). Physical activity at short term exacerbates decreased fat mass and has no protective effect on bone composition and lean mass (150, 211, 213, 316). When the protocol is maintained on the long term (55 days) like in FRW protocol, physical activity participates to body weight stabilization and to a significant slight body weight regain compared to pair-fed group (150). The long term protocol induces alterations in the bone mineral content leading in AN patients to osteoporosis. Indeed, in FRW mice, physical activity, currently described to stimulate bone formation, did not prevent on long term protocol the termination of bone mass acquisition induced by food restriction. Similar data were also described in SBA female mice subjected to a protocol of chronic stress associated with caloric restriction as previously mentioned. Such data confirmed the absence of protective effect of activity on bone mineral content in AN. In the ABA model, Pardo et al. (315) underline a differential tissue-specific expression pattern of ghrelin and leptin receptor at peripheral level reflecting tissue specific mechanisms to control energy homeostasis. The study of intestinal barrier indicates that the ABA protocol generates an increased colonic permeability associated with altered tight junction expression (309). These recent data open new windows to decipher the impact of gut microbiota in the deregulation of energy metabolism as well as the hepatic injury occurring in AN patients.

Besides alterations in various peripheral tissues, numerous endocrine changes are similar to that described in AN patients. Overall, ABA mice present lower plasma leptin and insulin concentrations and higher total plasma ghrelin and corticosterone concentrations (212, 215, 317). Moreover, energy metabolic factors are also changed in ABA/FRW mice with, in particular, an increase of free fatty acid and a decrease of glycemia (150, 213). On the long term, most of the endocrine alterations persist like lower plasma leptin concentrations, higher plasma total ghrelin concentrations still associated to lower glycemia, plasma ketone bodies, and higher free fatty acid in FRW mice (150). As highlighted previously, food restriction might induce shift in the energy metabolism regulations. Combination of food restriction and voluntary physical activity leads to a higher carbohydrate metabolism and a lower fat oxidation during the light period like the *ad libitum* control groups whereas at long term, FRW mice adopt a similar profile than the pair-fed group with a lipid metabolism more prominent. These changes point out the complexity of the peripheral regulation of nutrient and energy supplies, engaging probably hormones like leptin or ghrelin, which act on adipose tissues, muscles, or liver, might contribute to the changes/reduction in energy expenditure observed in FRW and pair-fed controls both at short and long term.

Central alterations are also observed in ABA protocols with an up-regulation of AgRP and NPY mRNA expression associated with a down-regulation of POMC and CART expression in the arcuate nucleus compared to control mice (211, 318–320). Surprisingly, no differences were observed concerning MCH and orexin expression in lateral hypothalamic area or CRH expression in paraventricular nucleus (211). However, until now, there is no study that evaluates potential changes in the expression of ghrelin and leptin receptors in ABA mice. Such information might be of importance since GHSR KO mice or intracerebroventricular injection of GHSR1a antagonist decreased the behavior of FAA and did not modify the food intake (321). Likewise, chronic subcutaneous or intracerebroventricular leptin injections lead to lower running wheel activity associated or not with reduction of food intake (322–324). Such fundamental researches are conducted to aim finding potential treatment using leptin or ghrelin to reduce hyperactivity frequently associated with AN, and leading to its excessive to emaciated phenotype. Indeed, intracerebroventricular injection of αMSH, whose release in hypothalamus is stimulated by leptin, enhances the ABA phenotype (325). Likewise, the specific sites of action of ghrelin and/or leptin in the ABA protocol should also be clarified. As an example, injections of ghrelin agonist in the lateral dorsal tegmental nucleus or its target, the ventral tegmental area, stimulate locomotor activity and food intake (326, 327). ABA mice are also shown to exhibit higher concentrations of noradrenaline, serotonine, but lower dopamine concentrations in the mediobasal hypothalamus compared to pair-fed and control groups (302, 328, 329). Moreover, Verhagen et al. (214) showed in the nucleus accumbens of ABA rats a lower circadian serotonergic activity without any changes for the circadian dopamine activity compared to control. These monoamines were suggested to play a role in voluntary food restriction in ABA rodents and in comorbidities observed in AN patients [i.e., depression or obsessive compulsive disorders; (137)]. It was suggested that reduction of physical activity is due to inhibition of serotonin release *via* 5HT1A autoreceptors in raphe nucleus (329–333). The opioid and endocannabinoid systems are also modified in the ABA model with increased plasma βendorphin concentration and pituitary βendorphin content in rats (334). This hyperendorphinism in the hypothalamo-pituitary-adrenal axis was linked to the auto-addiction hypothesis of AN. Furthermore, intraperitoneal injections of ABA mice with Δ9-tetrahydrocannabinol, an exogene ligand of cannabinoid receptors, increase their food intake, attenuate the body weight loss, reduce the energy expenditure, but increase the mortality rate compared to ABA mice vehicle-treated (308, 335). Due to the large distribution of endocannabinoid and opioid receptors in the brain, further studies are needed to clarify more precisely the mechanisms involved and the finely tuned interactions between all these homeostatic and non-homeostatic structures.

The ABA/FRW protocols also affect two other main endocrine functions: stress and reproduction. In ABA rodents, like in FRW mice (on the short and long term), a disruption of estrus cycle, vaginal closure, and reduction of ovaries size, and also hormone disturbances including a decrease of plasma testosterone and luteinizing hormone concentrations have been noted (150, 329, 336, 337). Reproduction axis is normalized when rodents are placed in recovery conditions, which reflect that reproductive disturbance is the result of energy unbalance (337). Concerning the HPA axis, ABA protocols induce on the short-term increased plasma corticosterone and ACTH concentrations and adrenal gland hypertrophy, but no significant modification of CRH expression in paraventricular nucleus compared to controls (211, 318, 338). Intracerebroventricular injection of CRH antagonist injection during the protocol leads to blunt the ABA phenotype (318). Furthermore, ABA adrenalectomized rats do not display increased wheel running activity (212). Once again, these data suggest that HPA axis is essential to apparition of ABA phenotype and point out the role of the glucocorticoids in the pathophysiology of AN. Somatotrope axis is another axis disrupted in AN patients, but in our knowledge there is no study using ABA protocol or associated protocols showing such alterations.

As mentioned earlier, one characteristic of the ABA model is the FAA. Several studies have documented the potential factors and neuronal structures leading to this particular behavior that can be generated like a foraging behavior or to increase the internal temperature due to energy deficit (212, 215, 321–323, 339). FAA itself can also influence the pattern of food intake. Indeed, in the FRW protocol, mice display a shift in the meal initiation compared to the pair-fed group (150). One explanation, suggested by Woods (340), considered eating to be a homeostatic stressful event, because the digested nutriments that reached the blood during and after a meal markedly disrupt energy homeostasis. Thus, the combination of both events, activity and feeding, could generate a stressful energy event especially in the short term, leading to increase in corticosterone levels and resulting to delay the meal initiation. Such phenomenon could occur in the ABA model, where the pattern of food intake has never been measured in metabolic cages, as it was done for FRW mice. The “self starvation” observed might thus be due to this delay in the initiation of the meal, which is, as mentioned above, time limited. Concerning the temperature, the ABA protocol induces a decrease of body temperature (341, 342). In addition, even if a negative correlation between FAA and body temperature was observed, no causal link has been demonstrated (325). Nevertheless, it was suggested that the decrease of body temperature is one of the factors contributing to physical activity (342). When ABA rats have access to a warm platform, they decrease their running wheel activity (320, 325, 343), similarly as observed in AN patients whose excessive physical activity vary depending on the ambient temperature (272).

All the data collected with both ABA and FRW models are totally useful to dissect the different mechanisms involved in the maintenance of the AN phenotype. Combining the different approaches on the short and long term will have an indubitable benefit to study the interactions between the various peripheral and central actors whose dialogs seem strongly impaired.

## Conclusion

**Figure 1:**
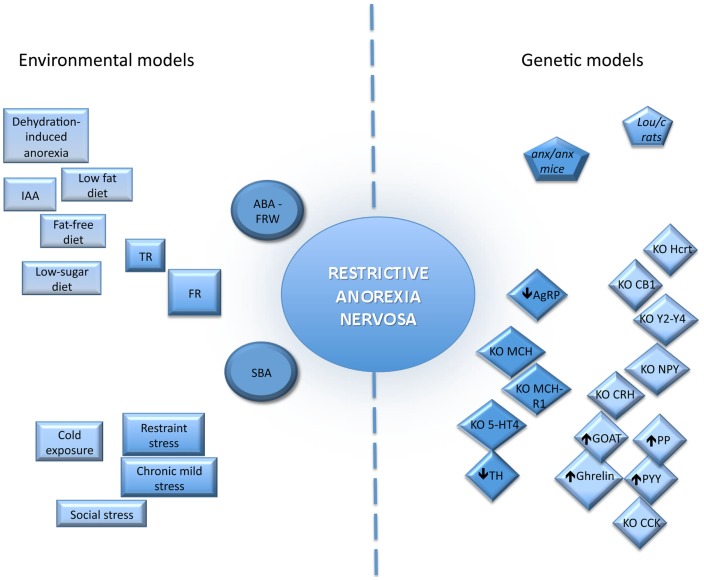
**Schematic representation of the relevance of the different animal models for anorexia nervosa, environmental and genetics, described in the review**. The more the models mimic restrictive anorexia nervosa symptoms the closer they are to the center of the figure. In the environmental models, the squares correspond to models based on one inducing factors and the circles to models based on several inducing factors. In the genetic models, the pentagons point out genetic models with spontaneous mutations whereas the diamonds show the genetically engineered mice: knock out (KO), transgenic (↑), specific deletion (↓). ABA, activity based anorexia; AgRP, agouti related peptide; CB1, cannabinoid receptor type 1; CCK, cholescystokin; CRH, corticotropin releasing hormone; FR, food restriction; IAA, indispensable amino acid deficient diet; GOAT, ghrelin O-acyltransferase; Hcrt, orexin/hypocretin; MCH, melanocortin concentrating hormone; NPY, neuropeptide Y; PP, polypancreatic peptide; PYY, peptide YY; SBA, separation based anorexia; TH, tyrosine hydroxylase; TR, time-restricted feeding; 5-HT R, serotonin receptor.

This review aims to depict the different animal models currently used or potentially interesting to study one or several aspects of restrictive AN (Figure 1). The definition of a pertinent animal model of psychiatric disorder remains extremely difficult. In the case of AN, more specially the restrictive subtype, many symptoms can be mimicked in rodents like the body weight loss, the changes in energy expenditure, increased physical activity, several endocrine and neurotransmitters changes that reflects similar physiological and neurobiological mechanisms inherent to the natural and adapted regulation of feeding. In this sense, some of the currently available animal models described here answer to the “face validity,” i.e., they mimic most of the symptoms of the human pathology. However, AN is usually associated with a refusal to eat. In rodents, such behavior is not natural, even if a kind of self-starvation is observed in migratory and hibernating animals. The “self-starvation” induced by some protocols does not reflect the human starvation, which is classically described to be associated with a personality trait involving neuronal inhibitory cognitive circuits. Even if self starvation is observed in some models like the well known ABA model, one may considered that the starvation is essentially due to physiological factors like temperature, dryness of the food, or even the delay in the initiation of the meal due to the intense physical activity observed before feeding. These models give certainly important information’s about the physiological changes occurring at this period, but do not reflect the self-starvation observed in human, which remains to be understood. Is it only driven by cognitive inputs or is it under the influence of factors regulating the feeding homeostasis like ghrelin or leptin which receptors are distributed in numerous “non-homeostatic” brain areas? Brain imaging might help to solve this question and would permit to give more credit to what we obtained in animal models. Even if all of these models do not fully answer to criterion of “construct validity,” i.e., a common etiology or similar conditions of induction, they fulfill the “predictive validity,” as the different pharmacological treatments used to restore body weight and other altered functions give encouraging results. As a conclusion, it is to note that current environmental models based on a combination of several inducing factors appear to be more relevant than the other models but may be to further improve studies on AN, new models coupling genetic and environmental factors remain to create and assess.

## Conflict of Interest Statement

The authors declare that the research was conducted in the absence of any commercial or financial relationships that could be construed as a potential conflict of interest.
